# Supplementary material for: Particle Morphology and Elemental Analysis of Lung Tissue from Post-9/11 Military Personnel with Biopsy-Proven Lung Disease
Source: Int J Environ Res Public Health. 2024 Jan 12;21(1):91. doi: 10.3390/ijerph21010091 (PMC10815659; doi:10.3390/ijerph21010091)
Supplement: Supplementary file 1 [file ijerph-21-00091-s001.zip › ijerph-2661963-supplementary.pdf]

S1 Supplemental Materials

Preparation of Tissue Samples for Analysis

Volume Calculations

The volume of each tissue scroll was calculated by first determining the area of the section as viewed on a hematoxylin and eosin (H&E) slide. This was accomplished using the magic wand tool of Adobe Photoshop to select the non-glass pixels. The area of the selected pixels was then multiplied by the 50 micron thickness to determine the volume. Overall, the volume of deployer tissue examined was more than the control tissue volume.

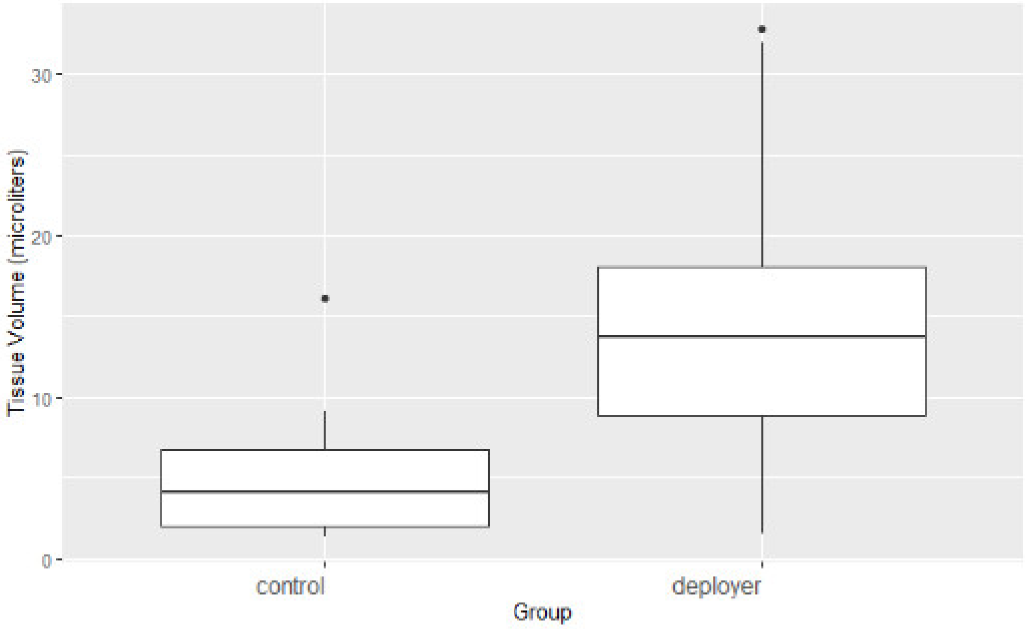

Supplemental Figure S1. Box plots of tissue volume of the control and deployer samples.

Trace Element Analysis

Hydrogen peroxide leachates/extracts: Aliquots of approximately 2.5 mL H_2O_2 extracts were evaporated gently at 100 °C in 4-mL Teflon vials (Savillex) on a Digi-Prep Cube (SCP Science) hot block to about 0.5-1 mL of liquid remained in the vials. The contents were then transferred to 2-mL microcentrifuge tubes. Teflon vials were rinsed with deionized water and added to the centrifuge tubes. Volume was completed to 2 mL with deionized water and then samples were centrifuged at 13,000 rpm for 30 minutes. After centrifugation, solution phase was gently transferred into 15-mL acid-cleaned Teflon vials (Savillex). A volume of 0.25 mL nitric acid (HNO_3, trace metal grade, Fisher Scientific) was added and contents were digested at 130 °C for 1 hour (closed-vessel digestion). After digestion, the contents were transferred to 15-mL acid-cleaned polypropylene test tubes. Volume was completed to 5 mL with deionized water (18.2 MΩ-cm). Final acidity of the samples was around 5% (v/v) HNO_3.

Acid-digestion of particulate matter fraction: After separating the H_2O_2-treatment fraction, 1 mL nitric acid (HNO_3, trace metal grade, Fisher Scientific) and 0.5 mL hydrochloric acid (HCl, trace metal grade, Fisher Scientific) were added to the same microcentrifuge tubes. Contents were shaken gently and allowed to react/dissolve for 30 minutes. Contents were then transferred to 4-mL Teflon vials (original sample containers) to combine with residual particulate matter. A volume of 0.5 mL perchloric acid ($HClO_4$, trace metal grade, Fisher Scientific) was added to the vials. Lids were closed and the contents were digested contents at 120 °C for 2 hours on a Digi-Prep Cube hot block (SCP Science). After digestion, contents were evaporated to near dryness at 120 °C to eliminate the excess acids. After evaporation, 0.25 mL of concentrated HNO_3 and 0.25 mL hydrofluoric acid (HF, trace metal grade, Fisher Scientific) were added to the vials. Lids were closed and contents were digested at 120 °C for 2 hours. At the end of the digestion, contents were cooled to room temperature and then transferred to 15-mL acid-cleaned polypropylene test tubes. Volume was completed to 5 mL with deionized water (18.2 MΩ-cm). Final acidity of the samples was around 5% (v/v) HNO_3 and 5% (v/v) HF.

Instrumental analysis: Elemental analysis of the prepared sample solutions was performed with ICP-MS. Solutions were directly analyzed by using a Perkin Elmer NexION 2000B ICP-MS instrument equipped with HF-inert sample introduction components, including a PFA spray chamber (Savillex), one-piece quartz torch with sapphire injector (Perkin Elmer) and a Teflon ARI-Mist nebulizer. All samples were

analyzed from undiluted solutions (5 mL volume). Instrumental operating conditions for ICP- are summarized below in **Supplemental Table 1**.

Supplemental Table S1. ICP-MS instrument operating parameters and conditions

Parameter	ICP-MS
RF power (W)	1500
Plasma argon flow (L/min)	13.0
Auxiliary argon flow (L/min)	0.85
Nebulizer flow (L/min)	0.86
Sample flow rate (mL/min)	0.5
Spray Chamber	PFA
Spray chamber temperature (°C)	Ambient ~ 20 °C
Scan mode	Peak hopping
Dwell time (ms)	30
Point/peak	1
Scans/peak	3
Scans/replicate	5
Measurement modes	KED and Standard

The ICP-MS instrument was calibrated with aqueous multielement standards in 2% HNO_3. Number of calibrants varied from 5 to 6 in addition to a calibration blank and required a R^2 of at least 0.995. Quality control/assurance for the instrumental determinations were carried out by analysis several USGS (T-series), and NIST water reference materials. These include T-215, 221 and 239, and SRM 1640 (Trace Elements in Natural Water). Sub-samples of the reference materials were analyzed by ICP-MS. Method blanks (n=6) were also prepared and analyzed for trace metal impurities for H_2O_2 treatments and particulate matter digestions. Instrumental concentrations for the unknown samples were corrected for method blanks to calculate the final elemental concentrations.

Comparing sequentially cut samples from the same lobe

To test the reproducibility of the sample, particle content of sequential slices from a lower lobe and upper lobe from two subjects were compared. Based on the SEM PM counts there is little consistency the between the 50 micron slices **(Supplemental Table 2).** ICP-MS results also show little consistency among the elements analyzed with the exception of Si and Al (**Supplemental Figure 2**).

Supplemental Table S2: Comparison of samples from the same lobe by category, count

Subject	1158 lower lobe			1025 upper lobe		
Sample	5003	5017	Difference	5004	5054	Difference
AlZr	15	15	0	55	1	54
C	26	581	555	768	746	22
Fe	119	462	343	85	713	628
GeoFe	29	39	10	12	39	27
Ti	40	21	19	39	2	37
Si	42	255	213	213	13	200
OSi	96	466	370	347	37	310
O	43	42	1	29	48	19

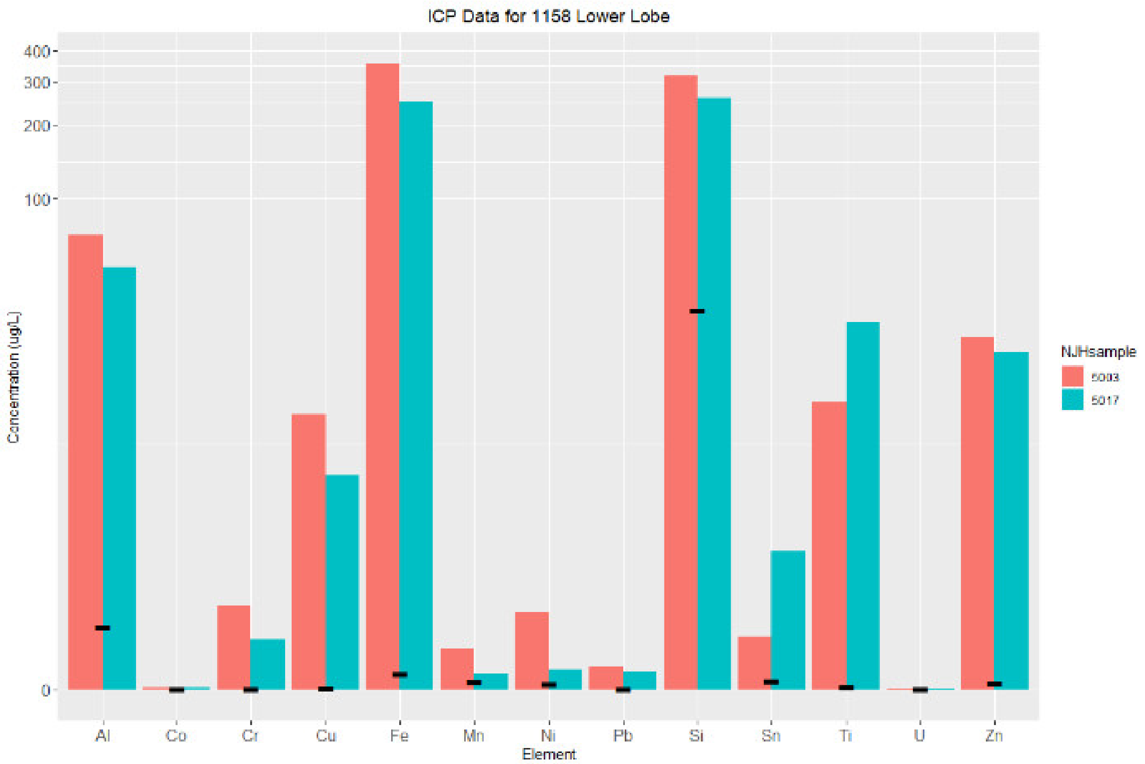

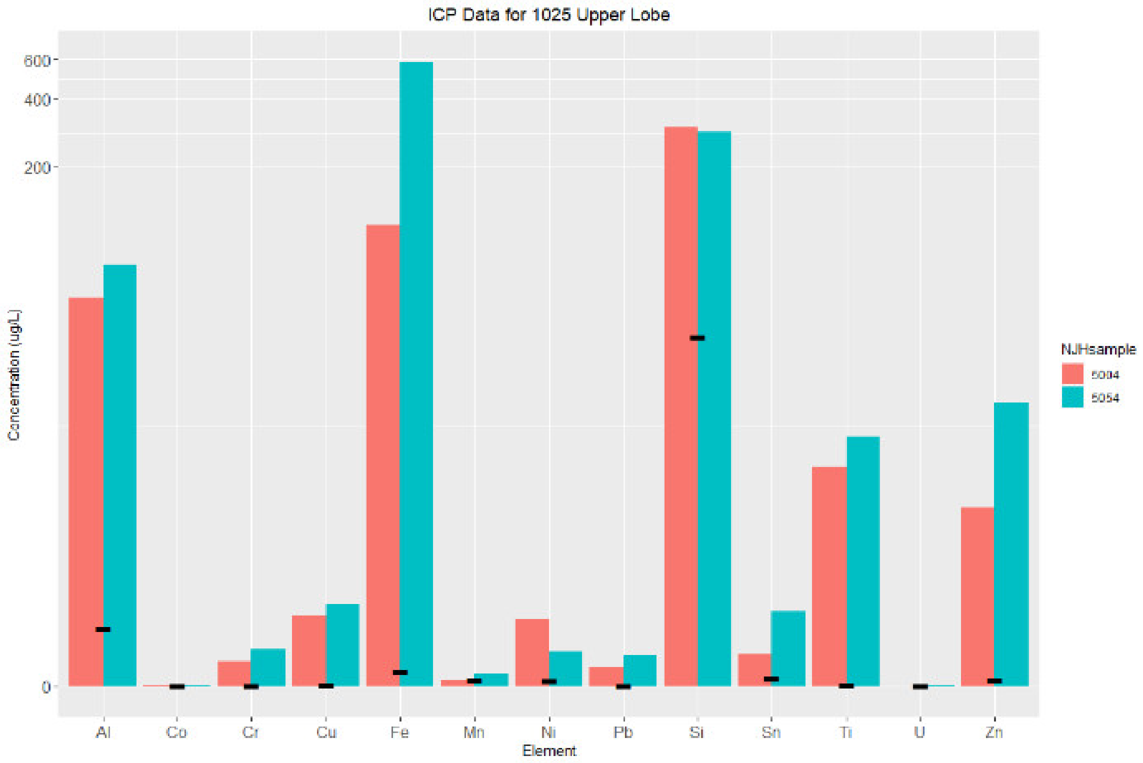

Supplemental Figure S2: Bar charts of the elemental concentrations from two serial slices (5003, 5017) taken from the lower lobe of subject 1158 (top) and two serial slices (5004, 5054) taken from the upper lobe of subject 1025 (top) same lobe. Concentrations are for PM + H_2O_2 fractions of the solution concentrations (µg/L). The black line for each element indicates the detection limit level.

Assessing Method Variability

To assess method variability, three samples with differing PM concentrations were re-analyzed for a total of three runs each. The repeat consisted of moving the field locations one over to account for loosely held particles that may have moved from previous electron beam exposure. We provide a summary of the spread of measured PM concentrations for each sample in **Supplemental Figure 3**. Each point represents a repeated run and each color represents a unique sample. Overall variability was largest for aluminum

and zircon oxides (AlZr). The average difference between the lowest and highest measurements for each PM category ranged from 1.5 to over 100 times (**Supplemental Table S3**).

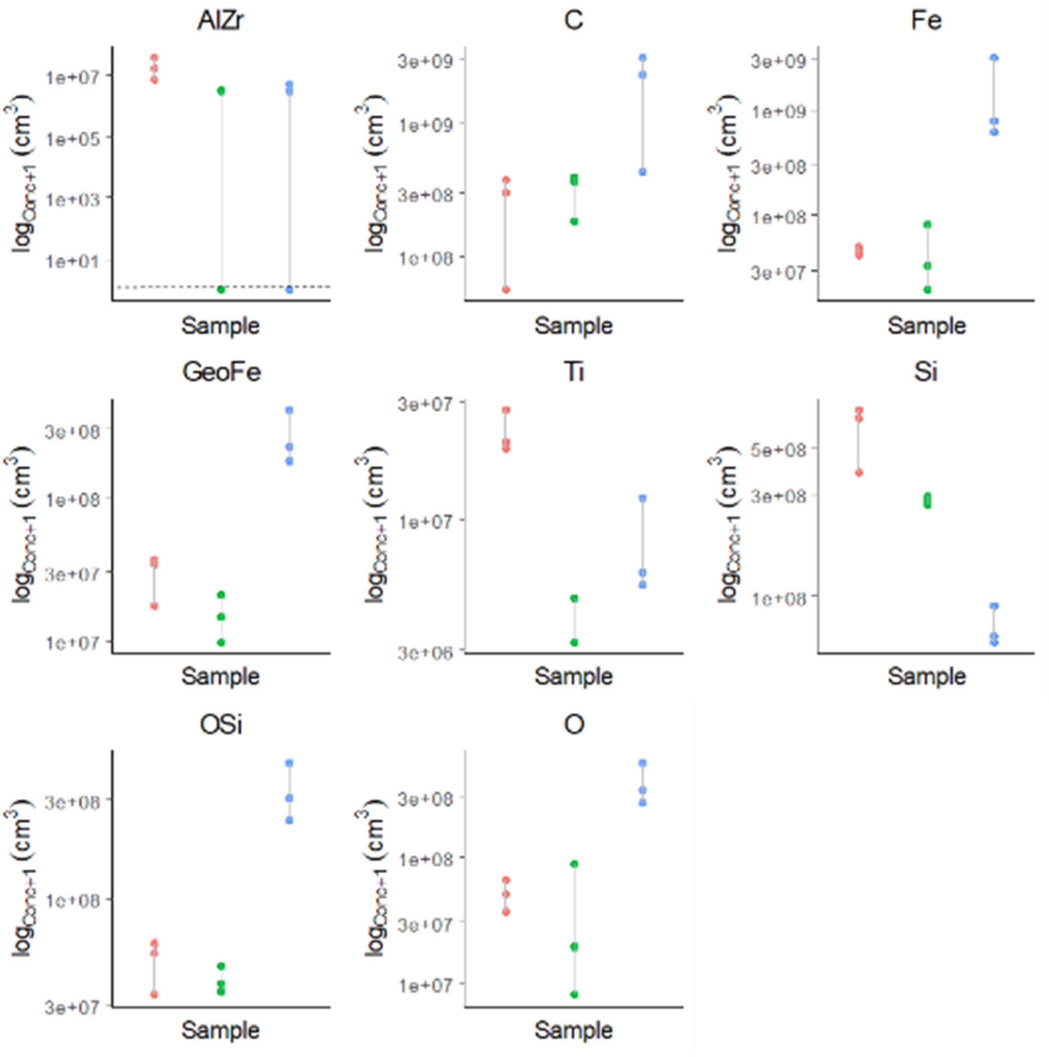

Supplemental Figure S3: Distribution of PM concentrations from three different samples (red, green, blue) by category for triplicate analytical analysis of the same sample: Aluminum and zircon oxides (AlZr), carbonaceous phases (C), iron (Fe), geogenic iron (GeoFe), titanium phases (Ti), silica (Si), other silicates (OSi), and other metals (O). Dashed line represents concentration of zero.

Supplemental Table S3: Average fold difference between the lowest and highest measurement for each PM category for three sample triplicates

Category	Average Difference
AlZr	>100*
C	4.7
Fe	3.0
GeoFe	2.2
Ti	1.7
Si	1.5
OSi	1.7
O	3.4

*For one run of two samples the minimum value was non-detected.

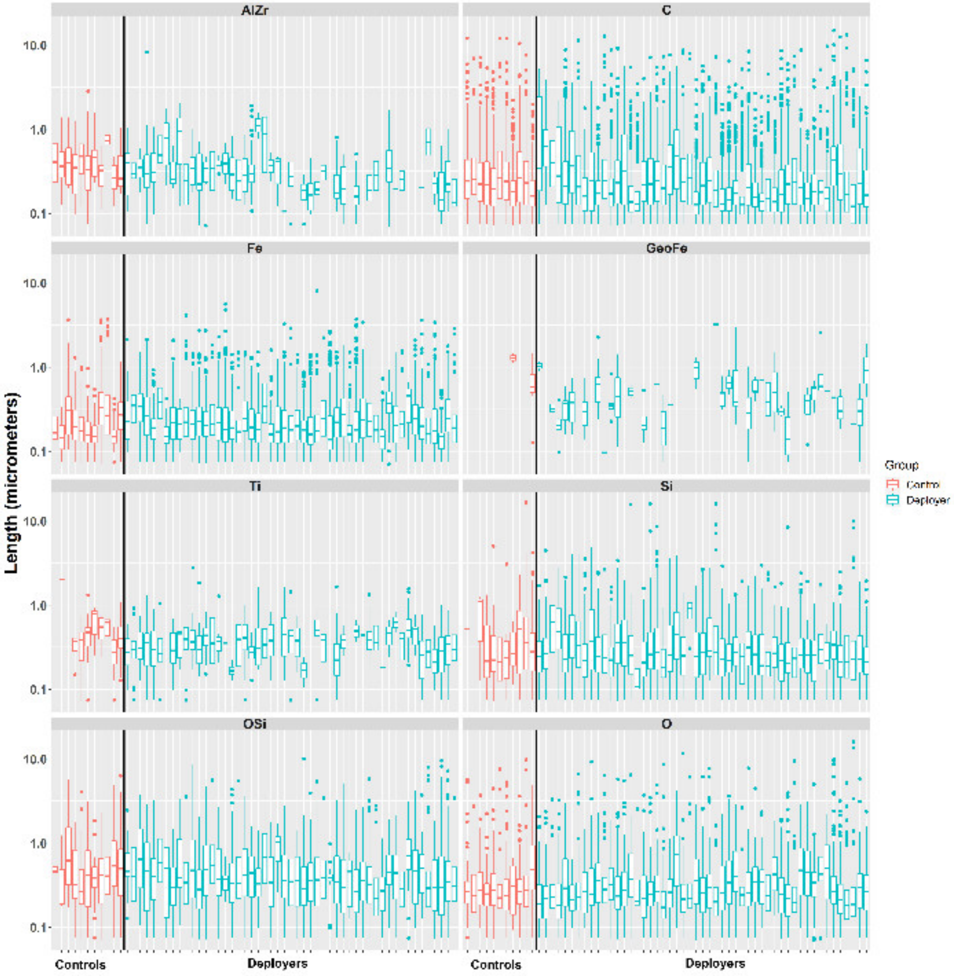

Supplemental Figure S4: Comparison of length of particles of aluminum and zircon oxides (AlZr), carbonaceous phases (C), iron (Fe), geogenic iron (GeoFe), titanium phases (Ti), silica (Si), other silicates (OSi), and other metals (O) between controls and deployers (separated by a solid black line). Each box plot represents a unique sample. While the PM lengths in deployers are generally smaller, there was no statistical difference for any PM category.

Supplemental Table S4: Estimated fold change and % change and 95% confidence interval comparing PM composition between deployers and controls

Category	Fold Change	95% CI	% Change	95% CI
AlZr	0.51	0.16, 1.63	-49%	-84%, 63%
C	0.62	0.40, 0.96	-38%	-60%, -4%
Fe	1.64	0.93, 2.90	64%	-7%, 190%
GeoFe	0.98	0.53, 1.82	-2%	-47%, 82%
Ti	3.85	1.08, 13.66	285%	8%, 1,266%
Si	**4.59**	**1.70, 12.39**	**359%**	**70%, 1,139%**
OSi	**3.36**	**1.58, 7.13**	**236%**	**58%, 613%**
O	0.62	0.33, 1.16	-38%	-67%, 16%

The composition of PM categories was compared between deployers and controls using a negative binomial mixed model with a random effect for each subject. PM classification count without blank correction was used. An offset was applied with the log of the total particle count for each sample. Comparisons were adjusted for age.

Supplemental Table S5: Estimated fold change and % change and 95% confidence intervals for the relationship between specific particulate matter (PM) categories or cumulative PM and deployment exposures (including weighted respiratory hazard scores and combat Military Occupational Specialty Codes [MOS])

Exposure	Change or Reference Group	Outcome	Fold Change	95% CI	% Change	95% CI	p-value
Sandstorm	Doubling	Proportion of Silica⁺	1.15	0.84, 1.57	15%	-16%, 57%	0.36
Sandstorm	Doubling	Proportion of Other Silicates⁺	0.98	0.75, 1.27	-2%	-25%, 27%	0.85
Burn pit	Doubling	Proportion of Metal Oxide⁺	1.04	0.85, 1.26	4%	-15%, 26%	0.71
Burn pit	Doubling	Proportion of Carbon⁺*	1.00	0.90, 1.10	0%	-10%, 10%	0.92
Total PM	Doubling	Total PM Burden^	1.02	0.90, 1.16	2%	-10%, 16%	0.76
Combat MOS	Non-combat MOS	Total PM Burden^	1.36	0.90, 2.03	36%	-10%, 103%	0.13

We used negative binomial mixed models (using PROC GLIMMIX) with a random effect for each subject and adjusted all analyses for age. We included either the log of the total particle count for each sample (⁺) as an offset or the log of the tissue volume and log of the fraction of the filter examined (^) as covariates in these models. We further adjusted comparisons of the carbonaceous PM fraction for reported diesel exhaust exposures (*).
